# RDDpred: a condition-specific RNA-editing prediction model from RNA-seq data

**DOI:** 10.1186/s12864-015-2301-y

**Published:** 2016-01-11

**Authors:** Min-su Kim, Benjamin Hur, Sun Kim

**Affiliations:** Interdisciplinary Program in Bioinformatics, Seoul National University, Seoul, Republic of Korea; Department of Computer Science and Engineering, Seoul National University, Seoul, Republic of Korea; Bioinformatics Institute, Seoul National University, Seoul, Republic of Korea

**Keywords:** RNA-editing, Condition-specific, Machine-learning, Random forest, RNA-seq, Systematic artefact

## Abstract

**Background:**

RNA-editing is an important post-transcriptional RNA sequence modification performed by two catalytic enzymes, "ADAR"(A-to-I) and "APOBEC"(C-to-U). By utilizing high-throughput sequencing technologies, the biological function of RNA-editing has been actively investigated. Currently, RNA-editing is considered to be a key regulator that controls various cellular functions, such as protein activity, alternative splicing pattern of mRNA, and substitution of miRNA targeting site. DARNED, a public RDD database, reported that there are more than 300-thousands RNA-editing sites detected in human genome(hg19). Moreover, multiple studies suggested that RNA-editing events occur in highly specific conditions. According to DARNED, 97.62 % of registered editing sites were detected in a single tissue or in a specific condition, which also supports that the RNA-editing events occur condition-specifically. Since RNA-seq can capture the whole landscape of transcriptome, RNA-seq is widely used for RDD prediction. However, significant amounts of false positives or artefacts can be generated when detecting RNA-editing from RNA-seq. Since it is difficult to perform experimental validation at the whole-transcriptome scale, there should be a powerful computational tool to distinguish true RNA-editing events from artefacts.

**Result:**

We developed RDDpred, a Random Forest RDD classifier. RDDpred reports potentially true RNA-editing events from RNA-seq data. RDDpred was tested with two publicly available RNA-editing datasets and successfully reproduced RDDs reported in the two studies (90 %, 95 %) while rejecting false-discoveries (NPV: 75 %, 84 %).

**Conclusion:**

RDDpred automatically compiles condition-specific training examples without experimental validations and then construct a RDD classifier. As far as we know, RDDpred is the very first machine-learning based automated pipeline for RDD prediction. We believe that RDDpred will be very useful and can contribute significantly to the study of condition-specific RNA-editing. RDDpred is available at http://biohealth.snu.ac.kr/software/RDDpred.

## Background

### RNA-editing: a biologically crucial regulator and highly condition-specific event

RNA-editing event is defined as a post-transcriptional RNA sequence modification [1]. Currently, there are two known RNA-editing mechanisms, performed by two different catalytic enzymes, “ADAR” (A-to-I) and “APOBEC” (C-to-U) [2, 3]. The most common type of editing in metazoans is the one catalyzed by the ADAR family of enzymes [4]. By utilizing high-throughput sequencing technologies, the biological function of RNA-editing has been actively investigated [5–7]. Currently, RNA-editing is considered to be a key regulator that controls various cellular functions including protein activity, alternative splicing pattern of mRNA and substitution of miRNA targeting site [1, 8–10].

Moreover, there are multiple studies that showed direct relation of RNA-editing to biological phenotypes. For example, Galeano’s group showed that the editing events in glioblastoma by ADAR2 enzymes are crucial for pathogenesis and claimed that ADAR-class enzyme can be considered as a tumor-suppressor [11]. And in APOBEC3G, a type of APOBEC-class enzyme causes HIV-1 retroviral inactivation by deamination [12].

**Fig. 1:**
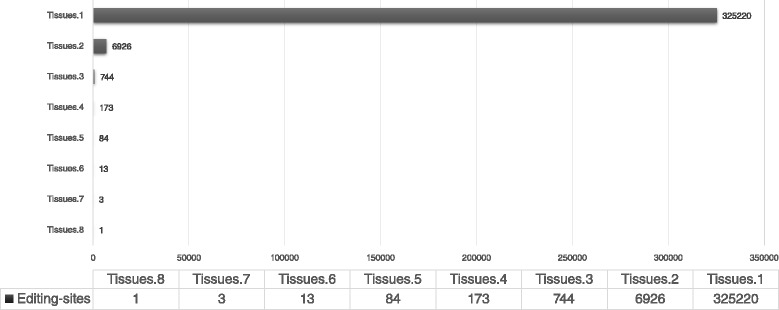
Low conservation rate with various tissues: In DARNED database [13], 97.62 % of total editing-sites are detected in a single tissue. Hence, showing significantly low conservation rate

DARNED, a well-curated public RNA-editing database have more than 300-thousands editing sites detected in the human genome hg19 [13]. Interestingly, the expression patterns of editing events in different conditions varied significantly. For example, in DARNED database, 333,164 editing sites in hg19 are registered from 21 independent studies in 139 tissues. The conservation rate among tissues is strikingly low, 97.62 % of these registered sites were detected from a single tissue or condition (Fig. 1). Moreover, multiple studies suggest that RNA-editing events can be involved in condition-specific regulation of genetic functions [14, 15]. Taken together, it is reasonable to believe that RNA-editing is highly condition-specific event.

### RNA-seq: an important tool for investigating condition-specific RNA-editing patterns

RNA-seq, a high-throughput sequencing of transcriptome, is a powerful method for investigating whole-transcriptome status. Since the nature of the technology is taking a snapshot of cells with massive sequencing reads, it is suitable for detecting condition-specific events in whole-transcriptome scale. Therefore, it is also suited for detecting RNA-editing events that have such condition-specific characteristics. There have been a number of studies that used RNA-seq to reveal condition-specific editing patterns in whole-transcriptome scale [5–7].

### Systematic artefacts: the major huddle to detect authentic RNA-editing events from RNA-seq

Even though RNA-seq is suitable for RNA-editing detection, it is also true that the current computational pipelines of RNA-editing detection with RNA-seq have considerable false-positive risks. In 2012 Nature Biotechnology journal, an article “The difficult calls in RNA editing”, reports interviews with eight prominent RNA-editing researchers. They pointed out that false-positive calling is one of the most challenging problems in RNA-editing detection with RNA-seq [16].

The false-positives caused by mis-alignment of short-reads can be termed “Systematic Artefacts” due to their inherent and reproducible characteristics. Systematic artefacts can be caused by various reasons, (a) inherent duplications/repeats within genomic sequences, (b) ambiguity caused by splicing-junctions, (c) prevalent polymorphisms between individuals and (d) shortness of sequencing reads [17, 18]. This inherent and reproducible error has been assumed to be one of the major confounding factors while detecting sequence variants [19, 20].

**Fig. 2:**
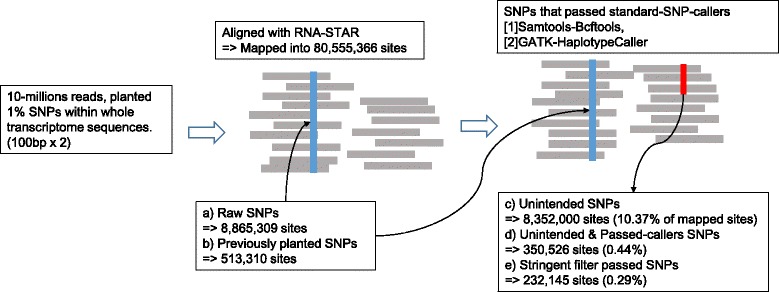
Substantial amounts of systematic artefacts: As simulation-test results, significant amounts of artefacts that consist of 0.29 % of total mapped sites are detected, which turned out to be indistinguishable even by the method combined with two state-of-art variant-callers and extensive *a priori* knowledges of genomic repeats

To assess the significance of confounding effects caused by systematic artefacts, we performed a simple simulation test in order to measure the false-detection rate of RNA-variants caused by mis-alignments. We used RNA-STAR, a state-of-art alignment tool in base-accuracy [20, 21], to evaluate inherent risks of false-positive detection in the human genome hg19 (Fig. 2). We simulated 10-millions reads from mRNA-seq [22] with 1 % simulated SNVs (513-thousand sites) and aligned them into the hg19 genome sequence. These 1 % of simulated SNVs are for representing the individual genetic differences including SNPs, somatic mutations, and RNA-editings.

**Fig. 3:**
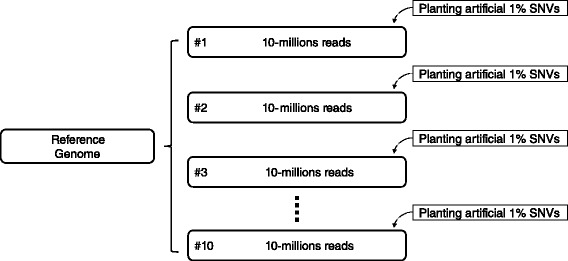
Simulation dataset preparation: We generated 10 simulated sequencing reads from spliced genomes (spliced by NCBI refseq annotation). Note that each reads are generated with different sets of SNVs

We generated 10 simulated data and aligned them (Fig. 3). In addition to the planted sites, we discovered that average 8.3 millions SNVs were detected from each 10-million reads(while we have planted 513-thousands sites). This is clearly artefacts. Such considerable number of unintended artefacts consist of 10.37 % of total mapped sites. Moreover, 2.78 % (232-thousands sites) of these unintended artefacts cannot be excluded by standard SNP-callers [23, 24] or by stringent filtering with error-inducible regions in hg19 genome [25] (Fig. 2). The result suggests that if we use 10-millions reads to detect RNA-editing, we will be confronted at least 232-thousands of artefacts that is difficult to be excluded by standard methods (Table 1).

**Table 1:** Artefact simulation results: resulted from 10-times of iterations

	Mapped reads	Mapped sites	Raw SNPs	Unintended SNPs	Caller passed	Filtered
Trial.1	9,734,787	80,552,288	8,872,433	8,358,426	350,694	231,972
Trial.2	9,735,558	80,558,479	8,878,304	8,365,007	350,670	232,496
Trial.3	9,733,473	80,568,898	8,880,681	8,366,553	350,136	231,912
Trial.4	9,733,159	80,570,416	8,879,502	8,365,311	350,442	231,539
Trial.5	9,733,939	80,545,810	8,853,408	8,339,822	350,332	232,008
Trial.6	9,733,507	80,542,007	8,838,870	8,326,074	350,917	232,649
Trial.7	9,734,222	80,555,307	8,859,741	8,346,628	350,390	232,128
Trial.8	9,735,046	80,562,701	8,874,369	8,361,655	350,807	232,063
Trial.9	9,733,971	80,555,609	8,852,720	8,339,866	350,059	232,336
Trial.10	9,734,717	80,542,143	8,863,065	8,350,655	350,809	232,347
Average	9,734,238	80,555,366	8,865,309	8,352,000	350,526	232,145

### Three distinct computational approaches that addressed the artefact-issue

To handle systematic artefacts in RNA-seq, a number of computational approaches have been developed. These can be categorized into three groups in terms of features they used: (a) *A priori* knowledge based filtering [26, 27], (b) Computational simulation of artefacts [6], (c) Machine-learning based prediction model [5, 28].

*A priori* knowledge based filtering used public genomic features, such as Alu repeats, genomic duplications, and pseudogenes, to assess the detected editing-sites directly. For instance, Li’s group used public annotation of genomic repeats to filter out potential artefacts within the detected RDD(RNA/DNA Difference) sites [26]. On the other hand, the approach based on computational simulation of artefacts rather utilizes calculated features than public features. Peng’s group used extensively simulated RNA-seq to predict inherent error-inducible regions in genome sequence and used them as a filter [6].

Unlike the filter-based methods that directly assess RDD candidate sites with pre-defined filters, machine-learning based methods generates a predictor in advance. The predictor, or machine-learning classifier, is trained to learn the differences between true and false examples. As an example, Laurent’s group generated a Random Forest predictor that utilizes read-alignment patterns as attributes. With 77 attributes, Laurent’s group generated a predictor and demonstrated it has 87 % of estimated accuracy by experimental validation [5]. As mentioned, since RNA-editing events are occurred highly condition-specifically, machine-learning approach might have an advantage in that they pursue more data-driven method by generating condition-specific model.

### Machine-learning based RNA-editing prediction became possible

Laurent’s work [5] was the first successful demonstration to show that a machine learning approach for RNA-editing prediction is both feasible and sensitive. However, to be a general-purpose model, there are several limitations. First of all, a predictor needs a training data that consists of positive and negative examples. And in Laurent’s study, they collected the both training examples from additionally performed Sanger-seq [5]. However, as we emphasized, RNA-editing is a condition specific event. And, since Laurent’s approach used experimentally verified training examples specific to their own conditions, the model might not be applicable in different conditions unless additional sequencing is performed. Therefore, it is more cost-efficient if we can avoid the experimental validations with utilizing the machine-learning approach.

## Methods

**Fig. 4:**
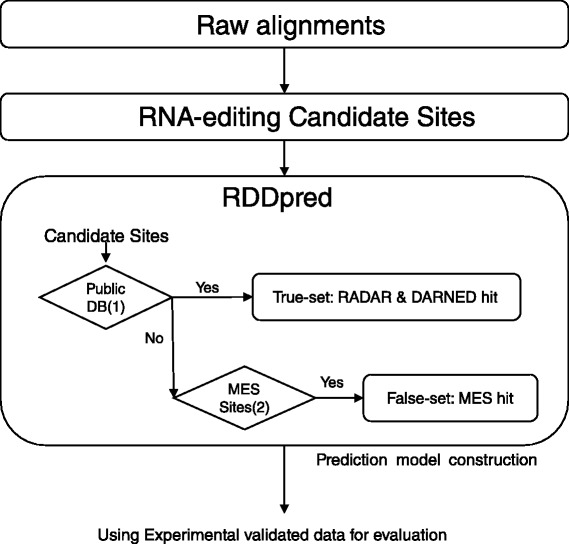
RDDpred workflow: 1) RDDpred takes raw alignments or raw RDDs 2) RDDpred arranges condition-specific training-data extracting positive/negative examples by mapping raw RDDs to public database (RADAR, DARNED) and MES-predicted sites, respectively. 3) RDDpred train the condition-specific classifier with arranged training-data and predicts true-editing against artefacts

Here we introduce RDDpred, a software package that is generally usable and do not need an experimental validation to prepare condition-specific training examples. Hence, RDDpred prepares condition-specific training data directly from input sequencing data or raw RDD candidates. In order to collect positive examples without experimental validations, we utilized two well-organized RNA-editing databases, RADAR and DARNED [13, 29]. Since we consider systematic artefacts as major cause of false-positives, we collect negative examples by utilizing the MES method that calculates the error-inducible regions within genome during alignments [6]. After collecting positive/negative examples from input data, all the remaining sites are considered as targets for prediction. RDDpred is a Random Forest predictor that utilizes 15 features that reflect the read-alignment patterns. The overall prediction scheme is illustrated in Fig. 4.

### Implementation of RDDpred

We tested RDDpred in Python (2.7.3), Samtools-Bcftools (1.2.1), WEKA (3.6.12) package, in linux environment.

### 1) Input and output of RDDpred

RDDpred takes alignment results as input data and gives the prediction results of each SNVs, or RDD candidates as outputs. The raw RDD candidates are detected with Samtools-Bcftools pipeline [23] while the prediction model is trained by using WEKA package [30].

### 2) Selection of alignment tool by the user

RDDpred can take inputs from any kind of alignment methods providing BAM-format outputs. However, we recommended RNA-STAR for its high degree of overall accuracy and ultra-fast performance [20, 21].

### Condition-specific training data preparation

### 1) Positive-set of training data: utilizing public databases, RADAR and DARNED

RADAR and DARNED databases include 2.5 million, 300-thousands of curated sites respectively [13, 29]. These two databases share a considerable portion of sites, 150-thousands sites. Since the pre-known sites are already proved to have editing potential, we can use the sites matched to the consensus sites as positive examples (Fig. 4). Since RDDpred takes the positive sites as an input, users can change or supplement the sites that are considered as true events.

### 2) Negative-set of training data: applying MES artefact calculation method

**Fig. 5:**
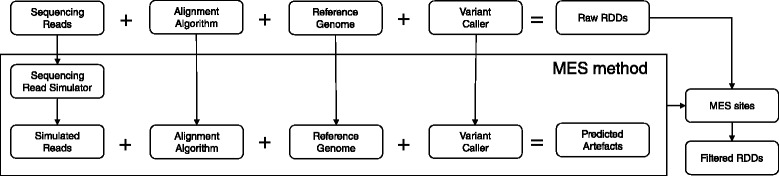
MES-calculation workflow: 1) MES method simulated randomly mutated sequencing reads. 2) Then, aligns them into genome sequences with an alignment tool of interest. 3) After the alignment finishes, uses variant-caller to detect raw SNPs. 4) Picks the SNPs sites that was not planted originally, i.e., unintended artefacts

To build a predictor, we also need negative examples. To address this issue, we utilized the MES method, a computational simulation for predicting error-inducible regions within genomes [6] (Fig. 5). With MES method, we can calculate error-inducible regions specific to the conditions, such as, SNPs combination of the samples, the experimental specification of sequencing, and the choice of alignment method. And if we calculate the error-inducible regions specific to our conditions, then we can consider them as potential error-prone regions. Therefore, just like collecting positive examples, we can reasonably assume the portion of RDD candidate sites that belong to those error-prone regions are probably systematic artefacts, thus using them as negative examples (Fig. 4).

### RDDpred predictor description

### 1) RDDpred mainly focuses on systematic artefacts

It is known that there are various types of artefacts from RNA-seq, such as amplification errors during library construction, sequencing errors, and errors by mis-alignments. Unlike former events, errors by mis-alignments shows different characteristics that they are more reproducible. Since the errors from library construction and sequencing procedures are transient in general, they can be excluded by replicating experiments. On the other hand, since the errors by mis-alignments, or systematic artefacts, are inherent to specific alignment method, they might not be excluded even after multiple replications. Therefore, RDDpred mainly focuses on detecting systematic artefacts with considering other artefacts as well.

### 2) Read-alignment pattern: a valuable source for distinguishing systematic artefacts

Read-alignment pattern is defined as local status of alignments in variant sites that has been utilized to distinguish artefacts [23]. There are at least six distinct categories of attributes calculated from read-alignments pattern. RDDpred utilizes 15 attributes of the six categories to generate a predictor, which are listed in Table 2. The six categories, such as "Read Depth", "Allele Segregation", "Mapping Quality", "Read Position", "Base Quality", and "Read strand" are known to have significant prediction power and also utilized by Samtools-Bcftools pipeline [23]. The attributes are basically measured as statistics to distinguish how the reads with variants are different with the reads without variants. RDDpred observed massive examples of positive/negative sites to learn how the statistics are represented differently between them. All of 15 attributes are calculated with Samtools-Bcftools pipeline during raw RDD detection [23]. Also we used WEKA, a data mining package, to train a prediction model [30]. Among the algorithms supported in WEKA, we chose Random-Forest algorithm, which showed the best performance in our evaluation datasets and showed significant performance in Laurent’s study [5].

**Table 2:** Attributes used to train prediction model: total 15-features are calculated with samtools-bcftools(v1.2) pipeline [23]

Categories	Attributes	Description
Read depth	Read depth	Read depth
Allele segregation	VAF	Variant read ratio
Allele segregation	SGB	Segregation based metric
Allele segregation	FQ	Phred probability of all samples
		being the same
Allele segregation	CallQual	Variant/reference QUALity
Mapping quality	PV3	Mapping quality bias
Mapping quality	MQB	Mann-Whitney U test of Mapping
		Quality Bias
Mapping quality	MQ0F	Fraction of MQ0 reads
Mapping quality	MQ	Root-mean-square mapping quality
		of covering reads
Read position	VDB	Variant Distance Bias for filtering
		splice-site artefacts in
		RNA-seq data
Read position	RPB	Mann-Whitney U test of Read
		Position Bias
Read position	PV4	Tail distance bias
Base quality	PV2	Base quality bias
Base quality	BQB	Mann-Whitney U test of Base
		Quality Bias
Read strand	PV1	Read strand bias

### 3) The 15 attributes for RDDpred

As mentioned, the 15 attributes are categorized into six category, (a) “Read Depth” category represents read-count in editing sites. (b) “Allele Segregation” category includes four attributes, such as VAF, SGB, FQ, and CallQual, respectively. All of these attributes are calculated from edited read-ratio against total reads. (c) “Mapping Quality” category of attributes reflects how the alignments of reads are well-performed, which utilizes alignment scores that the aligner generates. Four attributes, such as PV3, MQB, MQ0F and MQ belongs to this category. (d) “Read Position” category includes three attributes, such as VDB, RPB, and PV4, which represent how the positions of variants are biased within sequencing reads. (e) “Base Quality” category uses base-quality information generated by sequencing machine to detect whether low-quality bases are significantly biased to editing-sites. Two attributes, PV2 and BQB belongs to this category. (f) Finally, “Read Strand” category includes single attribute PV1, that represents how the strands of edited reads are biased than non-edited reads (Table 2).

## Results

### Evaluation with two previous studies

We evaluated RDDpred with two datasets from independent studies performed by Bahn’s and Peng’s group, respectively [6, 7]. Both studies computationally predicted RNA-editing sites and validated them with Sanger-seq. In Bahn’s study, RNA-seq produced 115,132,348 reads with 13,815,881,760 bases in human glioblastoma astrocytoma. RDDpred detected 6,856,440 raw RDDs from them and predicted 105,564 sites as true RNA-editings. In Peng’s study, RNA-seq produced 583,640,030 reads with 101,787,059,720 bases in human lymphoblastoid. In this case, RDDpred detected 58,666,976 raw RDDs from them and predicted 3,076,908 sites as true RNA-editings. Note that even though both study uses human tissues, they resulted different number of RNA-editing sites, 105,564 vs. 3,076,908, which indicates that the expression patterns of RNA-editing events might be different in two experimental conditions (Table 3).

**Table 3:** Comparison results from two different tissues which shows that RDD occurs condition specifically

	Reads	Bases	Raw RDDs	Accepted RDDs
Bahn’s	115,132,348	13,815,881,760	6,856,440	105,564
Peng’s	583,640,030	101,787,059,720	58,666,976	3,076,908
Fold	5.07	7.37	8.56	29.15

We constructed condition-specific model independently from each datasets and evaluated RDDpred separately with the corresponding validation results as test-data. And the test-data was the results of Sanger-seq by each groups. In the process of constructing each models, we carefully arranged the training-data in order not to contain any of information related to test-data. In other words, we did not use test-data for the construction of the model.

1. Training datasets
  - Positive examples: Predicted as positives sites by Public Databases (RADAR, DARNED)
  - Negative examples: Predicted as artefact sites by MES method (Peng et al. Nature biotechnology 2012)
  - The entries overlapped with test-data are excluded from training-data
2. Test datasets
  - Positive examples: Positively detected sites by experimental validation (Sanger-seq)
  - Negative examples: False discovery sites proved by experimental validation (Sanger-seq)

### RDDpred prediction in Bahn’s dataset

**Fig. 6:**
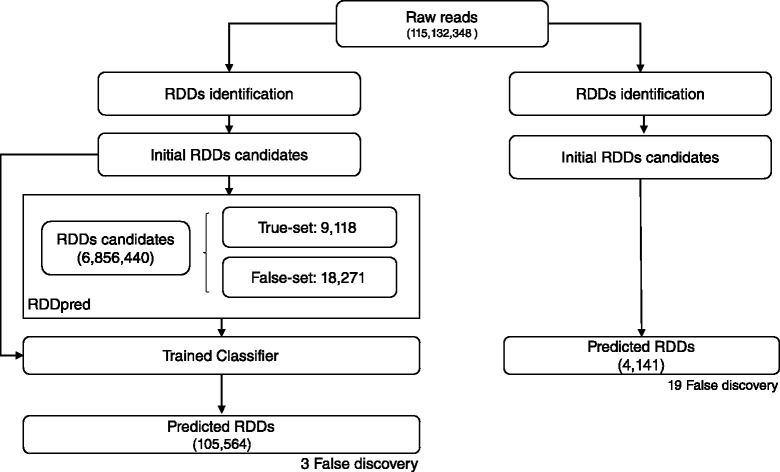
Overall prediction results: RDDpred predicted 105,564 sites from 6,856,440 initial RDDs candidates, showing that the model was able to reduce potential artefacts from initial RDD candidates with less false-discovery

**Fig. 7:**
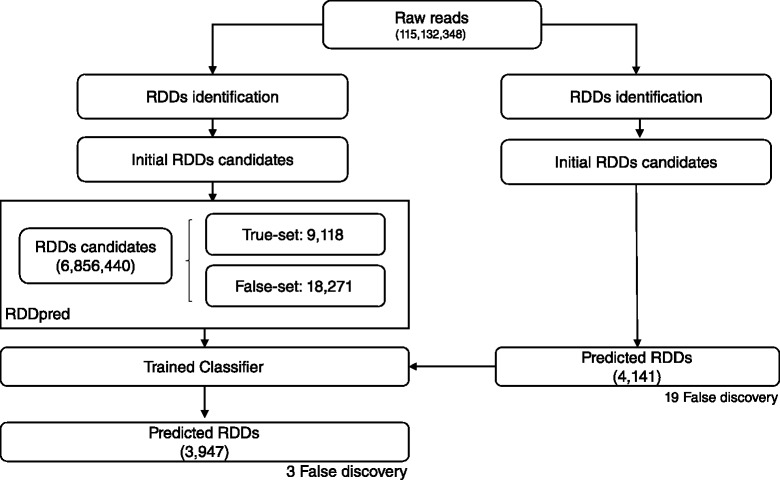
Comparison with Bahn’s prediction results: RDDpred predicted 3947 from 4141 sites indicating that 95.32 % of bahn’s results were successfully reproduced with less false discovery, resulting 84.21 % of NPV

RDDpred predicted 105,564 sites from 6,856,440 initial RDDs candidates, showing that not only the model was able to reproduce (95.32 %) the results but also was able to reduce potential artefacts (98.46 %) from Bahn’s study. Moreover, RDDpred have successfully reject most of the false discoveries in Bahn’s prediction. Resulting 84.21 % NPV(Negative Predictive Value). The following results indicate that RDDpred showed relative robustness than Bahn’s by reducing potential artefacts and rejecting false-discoveries (Figs. 6 and 7).

### RDDpred prediction in Peng’s dataset

**Fig. 8:**
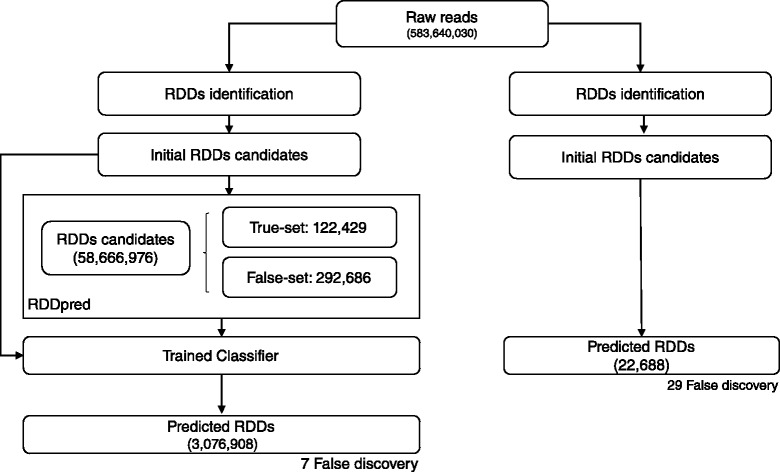
Overall prediction results: RDDpred predicted 3,076,908 sites from 58,666,976 initial RDDs candidates, showing that the model was able to reduce potential artefacts from initial RDD candidates with less false-discovery

**Fig. 9:**
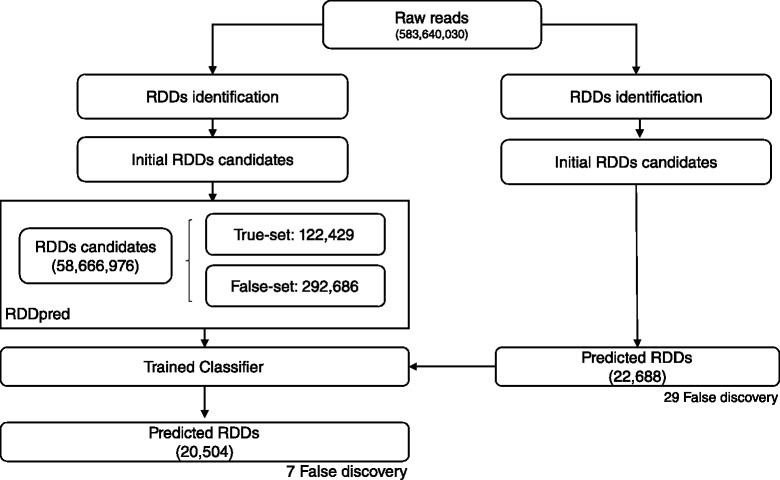
Comparison with Peng’s prediction results: RDDpred predicted 20,504 from 22,688 sites indicating that 90.37 % of bahn’s results were successfully reproduced with less false discovery, resulting 75.86 % of NPV

In Peng’s study, RDDpred predicted 3,076,908 sites from 58,666,976 initial RDDs candidates, which also proves that the model was able to successfully reproduce (90.37 %) the results but also reduce potential artefacts (94.79 %) from Peng’s study. Moreover, RDDpred have successfully reject most of the false discoveries in Peng’s prediction. Resulting 75.86 % NPV (Negative Predictive Value). The following results indicate that RDDpred also showed relative robustness than Peng’s by reducing potential artefacts and rejecting false-discoveries (Figs. 8 and 9).

### Additional specification of RDDpred

To provide information about actual running-time and memory-usage of RDDpred, we monitored the resource-usage while processing the Peng’s dataset. With the machine specified below, RDDpred took 18.33 hours to process 583,640,030 reads of 101,787,059,720 bases. We believe that the resources used here are not too excessive for common research groups.

1. Linux version: Linux version 2.6.32-358.el6.x86_64, CentOS release 6.4
2. Memory usage: 20 GB in maximum
3. CPU usage: 20-cores (Intel(R) Xeon(R) CPU E5645 @ 2.40 GHz)

## Discussion

We developed a software package for RNA-editing prediction from RNA-seq data. RDDpred utilizes current published database and methods such as RADAR, DARNED [13, 29] and MES-method [6] to build condition specific predictor. RDDpred generates a predictor that considers the experimental condition under which RNA-seq experiments are performed. As of now, there are only two studies we can compare with RDDpred. However, we successfully demonstrated that RDDpred was able to reproduce the results and reduce the false-discovery in both studies.

In order to investigate working principle of RDDpred in terms of prediction power of each categories, we calculated information-gain values with WEKA [30]. The rankings of six categories are listed in Table 4. Top 3 categories (“Read Depth”, “Allele Segregation”, and “Base Quality”) have strong prediction power. It is well known that “Read depth” or “Allele Segregation” categories of features are generally used for assessing the authenticity of variants [5, 7]. However, we discovered that the “Base Quality” category of attributes might also have significant prediction power according to the prediction results of two datasets.

**Table 4:** Category rankings of attributes utilized by RDDpred model: top 3 categories showed relatively strong prediction power

Category	DataA	DataB	RankA	RankB	RankMean
Read depth	0.4943	0.2515	1	2	1.5
Base quality	0.4498	0.40195	3	1	2
Allele segregation	0.457975	0.222825	2	3	2.5
Read position	0.195767	0.1019	4	4	4
Mapping quality	0.010025	0.047	6	5	5.5
Read strand	0.1584	0.0216	5	6	5.5

During the high-throughput sequencing, the sequencers generate bases-qualities that represent the confidence of sequencing. Therefore, unlike other five metrics, the “Base Quality” reflects the molecular status of bases that are directly recorded by sequencer. Until now, we only knew bases modified by editing enzymes are somehow recognized as guanine (or thymine for APOBEC class), but did not know how these recognitions are observed in the perspective of sequencing machines. The base-quality issue indicates that there might be some distinctions between normal and edited bases at the molecular level. Thus, it implies that more detailed recording of molecular characteristics during sequencing process might be a key to improve the accuracy of RNA-editing detection.

## Conclusions

### RDDpred: a useful tool for investigating condition-specific RNA-editing with RNA-seq

RNA-seq is one of the most powerful methods to investigate transcriptome and the amount of RNA-seq has recently increased nearly exponentially [31]. In spite of this rapid RNA-seq data accumulation and the recognition on important biological roles of RNA-editing, only a few studies reported RNA-editing findings due to the difficulty of getting robust profiles of RNA-editome [16]. Since it is difficult to perform the experimental validation of RNA-editing events in whole-transcriptome scale, a reliable and easily-usable prediction method is truly required.

RDDpred prepares training examples that are specific to the condition of input data without experimental validations. RDDpred proved good performances by reproducing the detection of two previous studies and correcting most of their false-discoveries. Moreover, as far as we know, RDDpred is the very first automated pipeline that utilizes machine-learning technique with a well-evaluated performance. Thus, we believe that RDDpred will be very useful and can contribute significantly to the study of RNA-editing. RDDpred is available at http://biohealth.snu.ac.kr/software/RDDpred.
